# Supplementary material for: A clinically identified Kirrel3 variant disrupts trans-synaptic circuit assembly
Source: bioRxiv. 2026 Oct 26:2026.10.20.760423. Preprint. [Version 1] doi: 10.64898/2026.10.20.760423 (PMC13733682; doi:10.64898/2026.10.20.760423)
Supplement: Supplementary file 1 [file NIHPP2026.10.20.760423v1-supplement-1.pdf]

bioRxiv preprint doi: https://doi.org/10.64898/2026.10.20.760423; this version posted October 26, 2026. The copyright holder for this preprint (which was not certified by peer review) is the author/funder, who has granted bioRxiv a license to display the preprint in perpetuity. It is made available under a CC-BY 4.0 International license.

Supplement to Figure 1

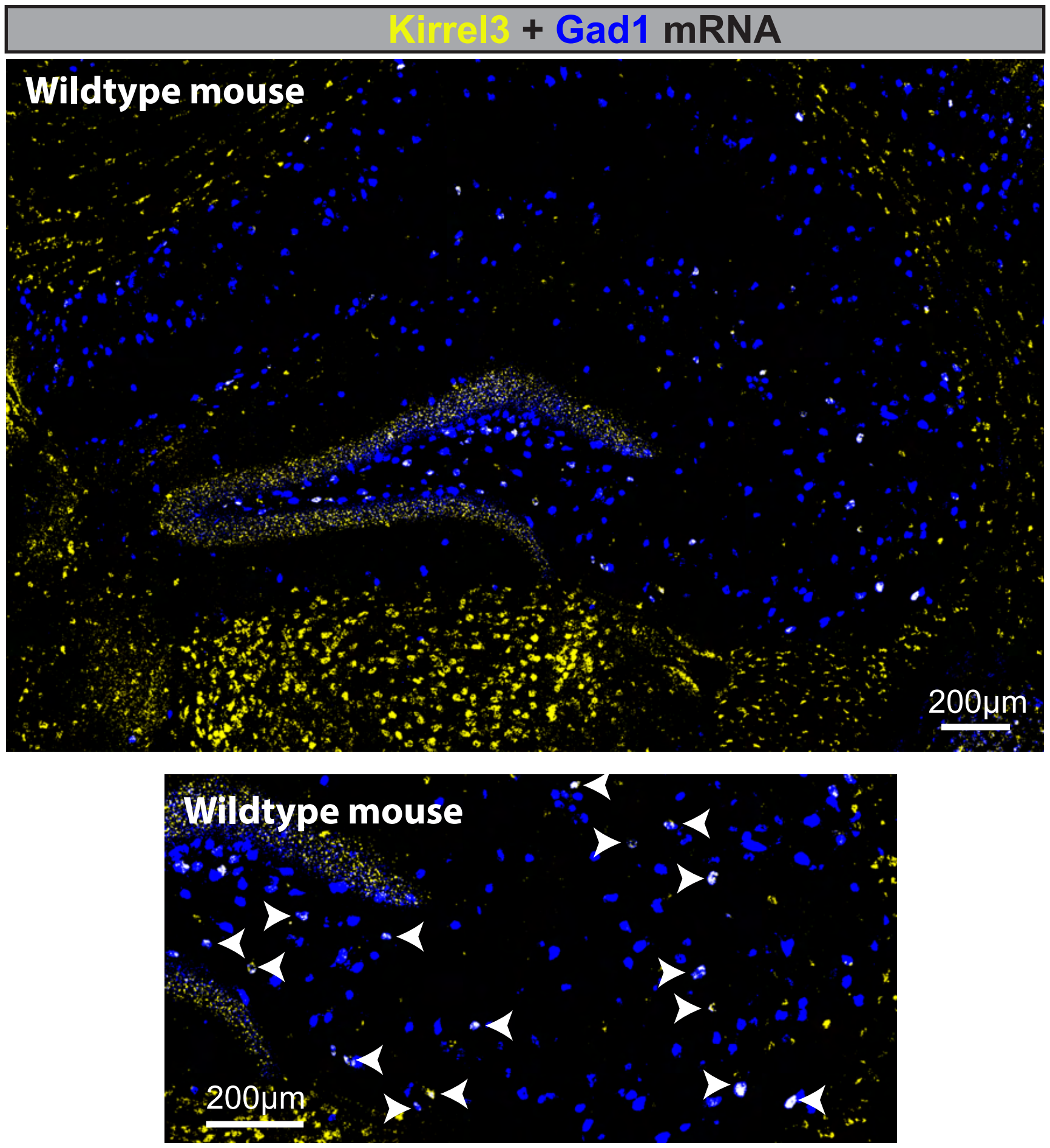

Supplement to Figure 1. Kirrel3 mRNA expression in wildtype hippocampus. Wildtype hippocampal section stained via fluorescence in situ hybridization for mRNA of Kirrel3 (yellow) and the GABA neuron marker Gad1 (blue). Bottom image is a magnified image showing scattered Kirrel3 expressing-GABA neurons indicated by the arrow heads.

bioRxiv preprint doi: https://doi.org/10.64898/2026.10.20.760423; this version posted October 26, 2026. The copyright holder for this preprint (which was not certified by peer review) is the author/funder, who has granted bioRxiv a license to display the preprint in perpetuity. It is made available under a CC-BY 4.0 International license.

Supplement to Figure 3

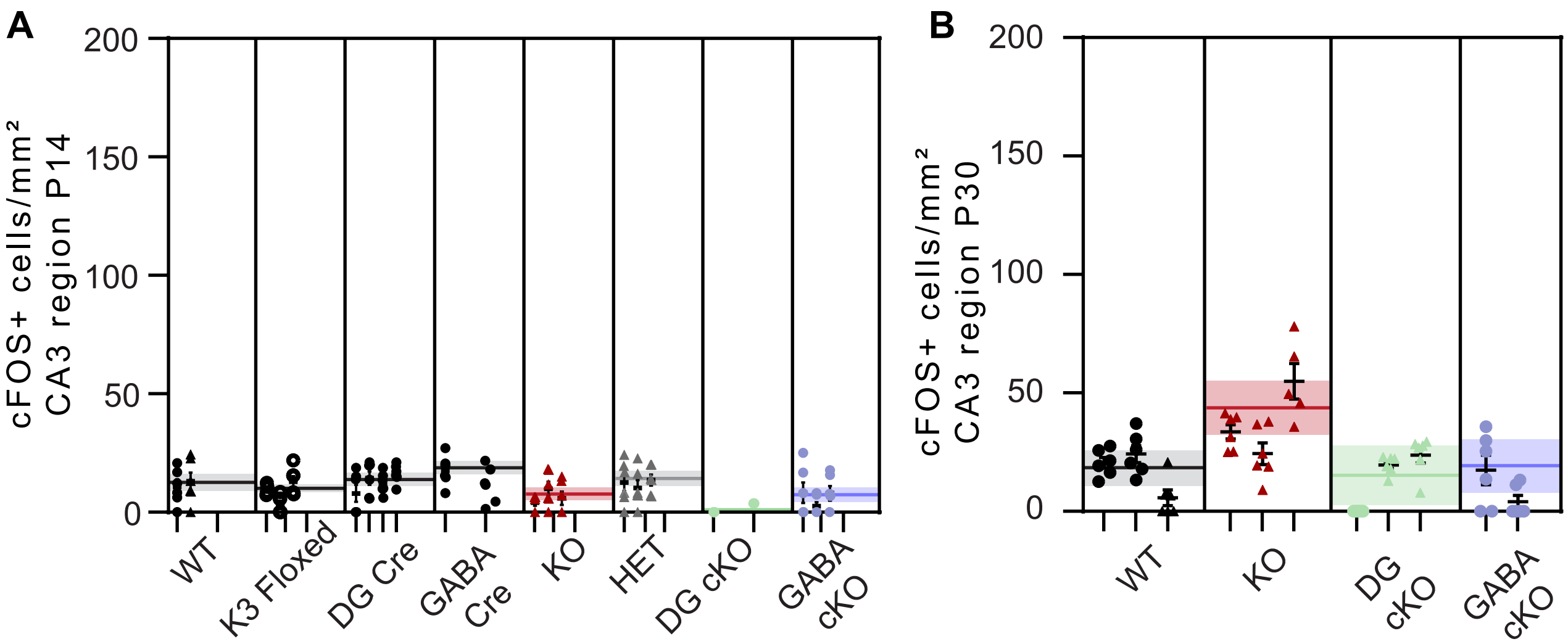

Supplement to Figure 3. CA3 cFOS quantification in the home cage condition. A-B) Quantification of the density of cFOS+ CA3 neurons in mice from each genotype that were perfused directly from their home cage (unstimulated) at P14 and P30. Each mouse is shown in a column with 6 brain sections (dots) per mouse. Males are represented by a triangle and females a circle. The horizontal line and shading across each genotype is the global average and SEM. Nested one-way ANOVA indicates a significant difference in A ($p=0.027$) but Dunnett's pairwise multiple comparisons indicate no significant differences for any genotype compared to WT control mice. ANOVA indicates no significant differences for B.

bioRxiv preprint doi: https://doi.org/10.64898/2026.10.20.760423; this version posted October 26, 2026. The copyright holder for this preprint (which was not certified by peer review) is the author/funder, who has granted bioRxiv a license to display the preprint in perpetuity. It is made available under a CC-BY 4.0 International license.

Supplement to Figure 5

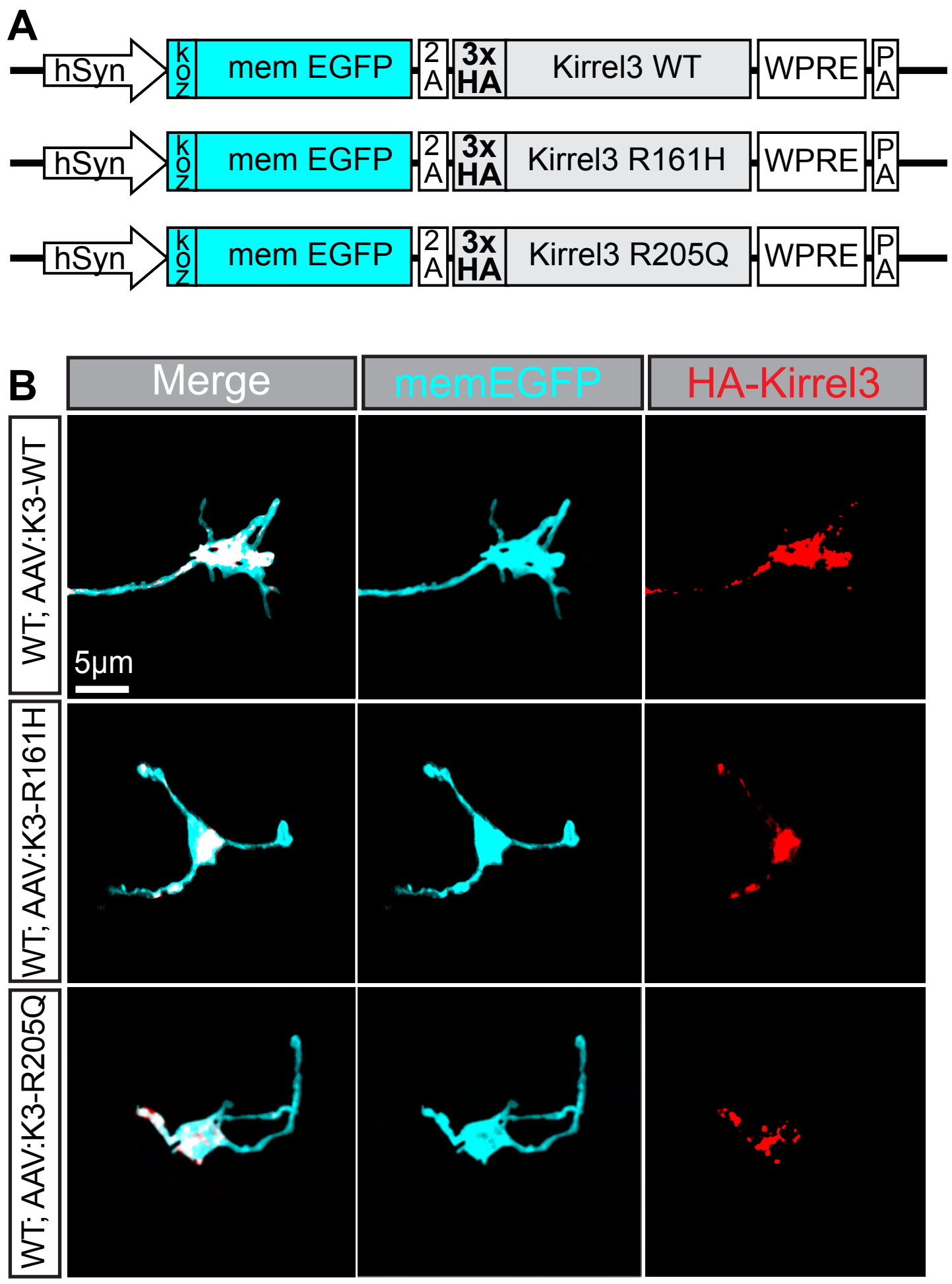

Supplement to Figure 5. Viral-mediated Kirrel3 expression in DG mossy fiber axons. A) Diagram of AAV constructs used to overexpress Kirrel3 wildtype or R161H and R205Q variants. All were made using the Kirrel3K isoform. **B)** Representative images of immunostained MFBs show the membrane GFP (cyan) and HA-tagged Kirrel3 (red) in the MFB and filopodia.

bioRxiv preprint doi: https://doi.org/10.64898/2026.10.20.760423; this version posted October 26, 2026. The copyright holder for this preprint (which was not certified by peer review) is the author/funder, who has granted bioRxiv a license to display the preprint in perpetuity. It is made available under a CC-BY 4.0 International license.

Supplementary Table 1: key resources

Reagent type (species) or resource	Designation	Reference	Identifier	Additional information
Strain, strain background (*Mus musculus*)	C57Bl/6J	The Jackson Laboratory	RRID:IMSR_JAX:000664	
Strain, strain background (*Mus musculus,* C57BL/6)	Kirrel3 floxed	[35]	NA	
Strain, strain background (*Mus musculus,* C57BL/6)	Dock10-Cre	[36]	RRID:MGI:6117432	
Strain, strain background (*Mus musculus,* C57BL/6)	Gad2-IRES-Cre	[37]	RRID:IMSR_JAX:010802	
Strain, strain background (*Mus musculus,* C57BL/6)	Kirrel3 knockout	[38]	NA	
Strain, strain background (*Mus musculus,* C57BL/6)	Ai3 Cre-dependent EYFP	[39]	RRID:IMSR_JAX:007903	
Instrument	Leica VT 1000S vibratome	Leica Biosystems	RRID:SCR_016495	
Instrument	LSM 980 confocal microscope	ZEISS	RRID:SCR_025048	
Antibody	goat anti-GFP	Abcam	#ab6673, RRID:AB_305643	IHC (1:2000)
Antibody	rabbit anti-GFP	Jackson ImmunoResearch Laboratories	#300-005-245, RRID:AB_3097738	IHC (1:2000)
Antibody	rabbit anti-cFos	Synaptic Systems	#226008, RRID:AB_2737414	IHC (1:500)
Antibody	goat anti-SPO	Santa Cruz Biotechnology	#51212, RRID:AB_1129844	IHC (1:5000)
Antibody	mouse anti-Kirrel3	Neuromab	#75-333, RRID:AB_2315857	WB (1:1000)
Antibody	mouse anti-GAPDH	Sigma Aldrich Millipore	#MAB374, RRID:AB_2107445	WB (1:2000)
Antibody	rabbit anti-HA	Cell Signaling	#3724S, RRID:AB_1549585	IHC (1:1000), WB (1:2000)
Antibody	mouse anti-FLAG	Sigma Aldrich Millipore	#F1804, RRID:AB_262044	WB (1:1000)
AAV (PHP.eB)	pAAV-CAG2-membrane (palmitoylation) EGFP	This paper	NA	MFB synapse assay
AAV (PHP.eB)	pAAV-Syn1-Lyn-EGFP-2A-3xHA-Kirrel3-isoformK-WT	Vector Builder	VB251010-1845akf	MFB synapse assay
AAV (PHP.eB)	pAAV-Syn1-Lyn-EGFP-2A-3xHA-Kirrel3-isoformK-R161H	Vector Builder	VB251010-1937rxm	MFB synapse assay

bioRxiv preprint doi: https://doi.org/10.64898/2026.10.20.760423; this version posted October 26, 2026. The copyright holder for this preprint (which was not certified by peer review) is the author/funder, who has granted bioRxiv a license to display the preprint in perpetuity. It is made available under a CC-BY 4.0 International license.

AAV (PHP.eB)	pAAV-Syn1-Lyn-EGFP-2A-3xHA-Kirrel3-isoformK-R205Q	Vector Builder	VB251010-1923eqm	MFB synapse assay
Software	ImageJ/Fiji	fiji.sc	RRID:SCR_002285	Image processing
Software	Zeiss Zen Blue v3.7 software	zeiss.com/microscopy/us/products/software/zeiss-zen.html	RRID:SCR_013672	3D MFB synapse analysis
Software	GraphPad Prism	graphpad.com	RRID:SCR_002798	Graph and statistics
Software	G*Power 3.1	psychologie.hhu.de/arbeitsgruppen/allgemeine-psychologie-und-arbeitspsychologie/gpower	RRID:SCR_013726	Power analysis

bioRxiv preprint doi: https://doi.org/10.64898/2026.10.20.760423; this version posted October 26, 2026. The copyright holder for this preprint (which was not certified by peer review) is the author/funder, who has granted bioRxiv a license to display the preprint in perpetuity. It is made available under a CC-BY 4.0 International license.

Supplementary Table 2: Statistics

Figure	Statistical test used	Group labels (G1-Gn)	#Treatments	#Subjects	#Values	overall nested ANOVA p value	F, DFn (degrees freedom) numerator, Dfd	Significance
2D	Nested one-way ANOVA	Floxed, DG cKO, GABA, cKO	NA	3,3,5	152	0.0025	14, 2, 8	yes
3C	Nested one-way ANOVA	Floxed, DG Cre, GABA Cre, DG cKO, GABA, cKO	NA	6,6,6,6,6	180	<0.0001	37, 4, 25	yes
3G	Nested one-way ANOVA	WT, KO, DG cKO, GABA cKO	NA	6,6,6,6	138	0.0056	5.7, 3, 20	yes
4B	Nested one-way ANOVA	WT, KO, HET	NA	4,5,4	156	0.0046	9.7, 2, 10	yes
4E	Nested one-way ANOVA	WT, KO, HET	NA	6,6,6	107	<0.0001	90, 2, 15	yes
6B	Nested one-way ANOVA	GFP, WT, R161H, R205Q	NA	3,4,6,6	188	0.0016	8.5, 3, 15	yes
6E	Nested one-way ANOVA	GFP, WT, R161H, R205Q	NA	5,6,6,6	137	<0.0001	15, 3, 19	yes
S3A	Nested one-way ANOVA	WT, Floxed, DG Cre, GABA Cre, KO, HET, DG cKO, GABA cKO	NA	2,3,4,2,3,3,2,3	132	0.027	3.3, 7, 14	yes
S3B	Nested one-way ANOVA	WT, KO, DG cKO, GABA cKO	NA	3,3,3,2	65	0.13	2.6, 3, 7	no
